# Orthotopic Heart Transplantation with Concurrent Coronary Artery Bypass Grafting Using In Situ Internal Thoracic Artery

**DOI:** 10.3390/jcdd13020092

**Published:** 2026-02-13

**Authors:** Arjun Verma, Andreas Habertheuer, Nikhil Prasad, Sameer Hirji, Michael M. Givertz, Jonathan W. Cunningham, Mandeep R. Mehra, Akinobu Itoh

**Affiliations:** 1 Division of Cardiac Surgery, Brigham and Women’s Hospital, Harvard Medical School, 75 Francis Street, Boston, MA 02115, USA; arjunverma@hms.harvard.edu (A.V.);; 2 Division of Cardiology, Brigham and Women’s Hospital, Harvard Medical School, 75 Francis Street, Boston, MA 02115, USA

**Keywords:** cardiac surgery, heart transplantation, coronary artery bypass grafting, marginal donor, organ allocation

## Abstract

Heart transplantation remains the definitive therapy for end-stage heart failure, but donor coronary artery disease (CAD) is a common reason for allograft refusal, limiting organ availability. We describe a case of orthotopic heart transplantation using a donor heart with isolated coronary artery disease managed with concurrent surgical revascularization. A 66-year-old male with end-stage non-ischemic cardiomyopathy requiring temporary mechanical circulatory support underwent heart transplantation using a donor allograft with a focal lesion in a large first diagonal artery. Following standard implantation, a left internal mammary artery–to–first diagonal artery bypass was performed prior to reperfusion. The patient was successfully weaned from cardiopulmonary bypass with improving left ventricular function and had an uncomplicated postoperative course aside from transient delirium and dysphagia. Echocardiography demonstrated recovery of normal left ventricular function, and the patient remained clinically well with preserved biventricular function at 7-month follow-up. This case demonstrates the feasibility of orthotopic heart transplantation with concurrent coronary artery bypass grafting using an arterial conduit and supports surgical optimization of select donor hearts, with focal coronary disease as a potential strategy to expand the donor pool without compromising short-term outcomes.

## 1. Introduction

Heart transplantation remains the gold standard treatment for end-stage heart failure; however, the scarcity of suitable donor organs remains a critical limitation [1]. Despite steady growth in the number of patients listed for transplantation, a substantial proportion of donor hearts are declined, often for potentially modifiable reasons. In response, transplant programs have increasingly adopted expanded donor criteria, with acceptable post-transplant outcomes reported for donors with advanced age, hepatitis C, and evidence of reversible ventricular dysfunction [2,3]. These strategies have resulted in a substantial expansion of the donor pool, with approximately one-fifth of transplants utilizing expanded criteria allografts [1].

The presence of donor coronary artery disease (CAD) is a major factor in allograft discard, with Guglin et al. noting a 32% prevalence among rejected hearts at their center [4,5]. Concerns surrounding donor CAD include the risk of early graft dysfunction, perioperative myocardial ischemia, and accelerated coronary allograft vasculopathy. Prior reports have described orthotopic heart transplantation with concurrent or staged coronary revascularization; however, these experiences are limited to small case series and remain heterogenous with respect to conduit selection and timing [6,7,8,9,10,11,12]. In this report, we describe a case of orthotopic heart transplantation with concurrent left internal mammary artery (LIMA) to first diagonal artery bypass for a discrete isolated coronary artery stenosis.

## 2. Case Report

### 2.1. Recipient

The patient is a 66-year-old male with end-stage heart failure secondary to non-ischemic cardiomyopathy. In 2010, he was found to have significantly depressed left ventricular dysfunction with an ejection fraction of 20% when he had palpitations. Over the next decade, he was essentially asymptomatic from a heart failure standpoint (New York Heart Association Class I–II). He did, however, suffer from recurrent ventricular tachycardia (VT), requiring catheter-based ablation, defibrillator implantation, and chronic amiodarone therapy. In May 2025, the patient presented to our hospital with acute decompensated heart failure. Given increasing pressor requirements and VT storms, he underwent percutaneous ventricular assist device (Impella 5.5, Abiomed, Danvers, MA, USA) placement and was listed for heart transplantation.

### 2.2. Donor

**Figure 1:**
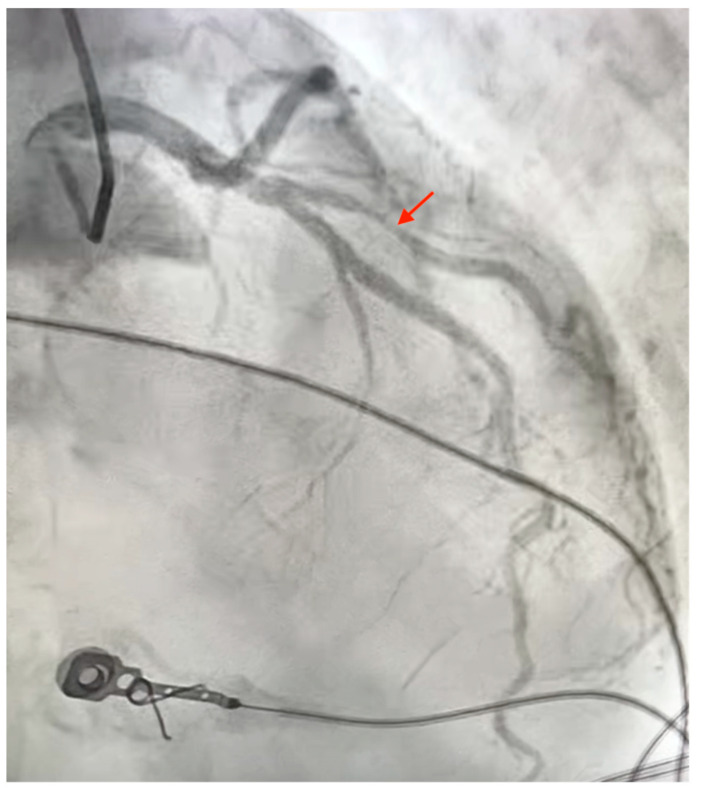
Suspected flow-limiting lesion in large first diagonal artery (red arrow).

Two weeks after listing, a donor heart became available from a 53-year-old male with no documented history of cardiovascular disease who suffered brain death following traumatic intracranial hemorrhage. Transthoracic echocardiography evaluation demonstrated normal biventricular function and competent valvular structures. Cardiac catheterization at the donor facility revealed a suspicious lesion in the proximal segment of a large first diagonal artery (Figure 1). Direct palpation and epicardial ultrasonography confirmed the presence of an isolated plaque in the first diagonal artery. No other detailed examinations, such as fractional flow reserve, intravascular ultrasound, or optical coherence tomography were performed. No other coronary abnormalities were detected. Given the large caliber of the vessel, the decision was made to proceed with surgical revascularization at the time of transplantation. Alternative strategies, including deferred percutaneous intervention or observation, were considered; however, concurrent bypass was favored to mitigate the risk of early graft dysfunction in the immediate post-transplant period and to avoid the utilization of antiplatelet therapy with percutaneous intervention.

### 2.3. Procedure

With the donor heart en route, the recipient’s LIMA was skeletonized and kept in situ. After recipient cardiectomy, the allograft was sewn to the recipient in the sequence of left atrium, aorta, and then the LIMA graft to the diagonal artery. The cross-clamp was removed and the heart was resuscitated. The rest of the anastomoses were then completed with the heart beating. The LIMA graft was found to have excellent flow with a minimal pulsatility index according to the flow measurement. The graft function recovery was satisfactory. Total cardiopulmonary bypass time was 175 min, with 76 min of cross-clamp time and 58 min of warm ischemic time.

### 2.4. Postoperative Course and Follow-Up

The patient left the operating room on low dose inotropic and vasopressor support. He was extubated on postoperative day 1 and transferred to the general ward on postoperative day 6. His recovery was delayed due to delirium and dysphagia, but he was discharged to an inpatient rehabilitation facility on postoperative day 39 with the transthoracic echocardiography demonstrating normal left ventricular systolic function (EF 55%). At 7-month follow-up, the patient is doing well and has preserved biventricular function. Given that the patient has remained asymptomatic, post-transplant coronary angiography or computed tomography angiography has not been performed.

## 3. Discussion

Efforts to expand the donor pool for heart transplantation have typically involved liberalization of standardized donor criteria and use of so-called “marginal donors” [1,13]. While these strategies have increased organ utilization, a substantial proportion of allografts continue to be declined for potentially modifiable reasons, particularly donor coronary artery disease.

Direct allograft optimization through surgical intervention is a less-described strategy for increasing access to suitable donor organs [14,15]. Thomson and colleagues reported the first case of orthotopic heart transplantation (OHT) with concurrent coronary artery bypass grafting (CABG) in 1988 [16]. Additional case reports and limited series have been published in the decades thereafter [6,7,8,9,10,11,12].

### 3.1. Choice of Conduit

As shown in Table 1, there is considerable variation in the utilization of arterial and venous conduits for simultaneous OHT and CABG. In patients undergoing isolated CABG, arterial conduits are associated with superior long-term graft patency compared with saphenous vein grafts [17,18]. However, these data must be cautiously extrapolated to the transplant population. Saphenous vein grafts may be considered in the transplant setting for several reasons. From a technical standpoint, the distal and proximal anastomoses may be completed on the back table prior to implantation, thereby limiting warm ischemia time [12]. Moreover, operative time for the recipient is reduced as there is no need for LIMA takedown. On the other hand, it is important to note that an arterial conduit can be utilized with acceptable bypass and warm ischemia times, as noted in this case. This case report illustrates the feasibility of arterial conduit use when anatomy, donor characteristics, and operative conditions are favorable. Additional cases with arterial or vein conduits are warranted to assess the superiority of arterial versus vein conduit for allograft coronary artery disease.

### 3.2. Cardiac Allograft Vasculopathy

An important consideration is the differential risk of coronary artery vasculopathy (CAV) for arterial and venous conduits. Some have suggested that CAV predominantly affects the arterial system, and thus, saphenous vein grafts should be utilized for revascularization during OHT. However, microscopic tissue examination by Oni et al. demonstrate that CAV also alters the venous system of the heart such as intimal hyperplasia and thickening [19]. These data call into question the theoretical advantage of vein grafts within the post-transplant inflammatory milieu [20,21].

## 4. Conclusions

This case demonstrates that orthotopic heart transplantation with concurrent surgical revascularization using an arterial conduit is technically feasible and can be associated with favorable short-term clinical outcomes. While longer-term angiographic follow-up and larger series are required to define durability, patency, and CAV-related outcomes, targeted surgical optimization of donor hearts with focal CAD may represent a viable strategy to safely expand the donor pool in carefully selected patients.

## Figures and Tables

**Table 1:** Previously published case series discussing orthotopic heart transplantation with concurrent coronary artery bypass grafting. LIMA: Left Internal Mammary Artery; SVG: Saphenous Vein Graft.

Author	Sample Size	LIMA:SVG	Follow-Duration (Months)	Survival	Patency
Laks et al. 1993 [6]	4	4:0	-	-	-
Rao et al. 2000 [7]	1	1:0	16	100%	100%
Abid et al. 2002 [8]	4	4:0	42	100%	75%
Marelli et al. 2003 [9]	12	1:23	24	85%	82%
Musci et al. 2004 [10]	2	0:2	40	100%	100%
Pinto et al. 2013 [11]	1	1:0	36	100%	100%
Yang et al. 2014 [12]	11	0:13	32	72%	-

## Data Availability

No new datasets were generated or analyzed beyond the clinical information presented within the manuscript itself.
